# Low‐Energy Electronic Excitations of N‐Substituted Heteroacene Molecules: Matrix Isolation Spectroscopy in Concert with Quantum‐Chemical Calculations

**DOI:** 10.1002/chem.201903371

**Published:** 2019-10-30

**Authors:** Jean Thusek, Marvin Hoffmann, Olaf Hübner, Olena Tverskoy, Uwe H. F. Bunz, Andreas Dreuw, Hans‐Jörg Himmel

**Affiliations:** ^1^ Anorganisch-Chemisches Institut Ruprecht-Karls-Universität Heidelberg Im Neuenheimer Feld 275 69120 Heidelberg Germany; ^2^ Interdisziplinäres Zentrum für Wissenschaftliches Rechnen Ruprecht-Karls-Universität Heidelberg Im Neuenheimer Feld 205 69120 Heidelberg Germany; ^3^ Organisch-Chemisches Institut Ruprecht-Karls-Universität Heidelberg Im Neuenheimer Feld 270 69120 Heidelberg Germany

**Keywords:** acenes, aggregation, electronic structure, heterocycles, matrix isolation

## Abstract

N‐Heteropolycycles are attractive as materials in organic electronic devices. However, a detailed understanding of the low‐energy electronic excitation characteristics of these species is still lacking. In this work, the matrix isolation technique is applied to obtain high‐resolution absorbance spectra for a series of tetracene and core‐substituted N‐analogues. The experimental electronic excitation spectra obtained for matrix‐isolated molecules are then analysed with the help of quantum‐chemical calculations. Additional lower energy excitation bands in the spectrum of the core‐substituted N‐derivatives of tetracene could be explained in terms of intensity borrowing from dipole‐forbidden transitions due to Herzberg–Teller vibronic coupling. In the case of tetracene, evidence for the additional formation of London dimers (J aggregates) is found at higher tetracene concentrations in the matrix.

## Graphical abstract

**Step into the matrix**: N‐Heteropolycycles are attractive as materials in organic electronic devices, but there is a lack of a detailed understanding of the low‐energy electronic excitation characteristics of these species. The matrix isolation technique enables detailed analysis of low‐energy electronic transitions of tetracene and N‐substituted tetracenes.

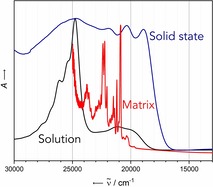

## Introduction

Acenes are linear polyaromatic hydrocarbons formally composed of annulated benzene rings.^1^ They have been attracting interest from both theoretical and experimental chemists for nearly a century.^2^, ^3^, ^4^, ^5^ In recent decades, polycyclic aromatic hydrocarbons, in general,^5^, ^6^, ^7^ and acenes, in particular,^8^, ^9^, ^10^, ^11^ have gained attention as potential materials in organic electronic devices due to their low and tuneable optical and electronic properties. More recently, N‐heteroacenes^12^ have started to play a significant role in materials research due to their improved solid‐state packing^13^ and more effective charge‐transport properties compared with those of the unsubstituted acene analogues.^11^, ^14^ They have been suggested as promising n‐type semiconducting materials,^15^, ^16^ with high electron affinities and small reorganisation energies, in which CH−N interactions change the packing from a herringbone‐like to a graphite‐like structure.^17^

**Scheme 1:**
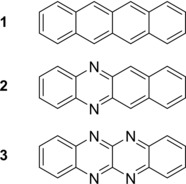
Overview of the substances studied herein.

The smaller members of the acene family have been extensively studied and are therefore well known in the literature.^18^, ^19^, ^20^, ^21^, ^22^, ^23^, ^24^, ^25^, ^26^, ^27^ Pioneering studies on side‐chain‐substituted acene derivatives by Anthony et al. have extended the range of stable, and therefore, synthetically accessible members of the acene family to longer members.^8^, ^28^, ^29^ Following newer thermo‐ or photochemical approaches to unsubstituted larger acenes,^30^, ^31^, ^32^ starting from pentacene, as well as matrix‐assisted in situ generation techniques by Bettinger et al.,^33^, ^34^, ^35^, ^36^, ^37^, ^38^ the spectroscopic characterisation of larger acenes, up to undecacene, has also become accessible. Some heteroaromatic derivatives of tetracene (**1**), such as benzo[*b*]phenazine (**2**)^39^ or quinoxalino[2,3‐*b*]quinoxaline (**3**; Scheme 1),^40^ have been investigated in this respect as well.

The matrix isolation technique with noble gases is a powerful tool for studying the electronic structure and molecular properties to minimise extrinsic effects, such as solvation or aggregation.^41^, ^42^, ^43^, ^44^ It allows for the application of standard spectroscopic methods, for example, IR, UV/Vis or Raman scattering measurements, that are not readily applicable in the gas phase. The most useful features of absorbance measurements on substances trapped in solid noble‐gas matrices include narrow band widths and the avoidance of hot bands.

However, for N‐substituted heteroacenes, a matrix‐based analytical study of electronic absorbance spectra has, to the best of our knowledge, not yet been reported. This work aims to provide a comprehensive comparative overview of electronic absorbance spectra of a series of members of the four‐membered acene and N‐heteroacene family, that is, **1** and the core‐substituted analogues **2** and **3** (Scheme 1). A comparison between electronic absorbance spectra of solutes, the solid state and substances trapped in solid neon matrices is presented. The vibrationally resolved electronic matrix spectra were simulated and analysed with the help of quantum‐chemical calculations, as described in more detail in the Experimental Section.

## Results and Discussion

### Comparison of UV/Vis methods

**Figure 1:**
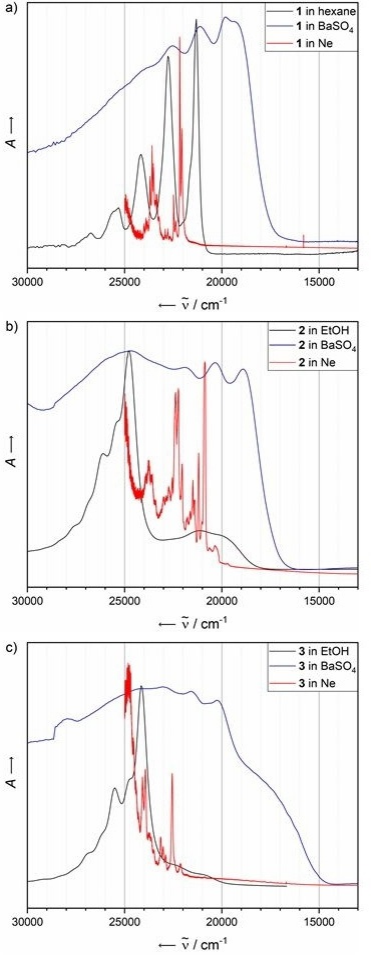
A comparison of UV/Vis spectra of a) **1**, b) **2** and c) **3** obtained by using different methods. Black: Transmittance of a 10^−5^ m solution in ethanol or hexane. Blue: Solid‐state reflectance spectrum of a BaSO_4_ matrix; the absorbance has been calculated as −log *τ*. Red: Visible spectrum of the isolated acene trapped in solid Ne at 4 K.

We have conducted comparative studies of UV/Vis measurements by using three different methods: usual transmittance measurements of solutions of the respective substance with concentrations of 10^−5^ mol L^−1^, solid‐state diffuse reflectance measurements of a matrix of the respective acene in BaSO_4_ (1:5) and visible measurements of the acene trapped in solid Ne at 4 K. All absorbance spectra are summarised in Figure 1.

The absorbance characteristics of **1** are similar in all studied environments. All spectra have distinct signals, with a vibrational progression of about 1400 cm^−1^. In the case of heteroaromatic compounds **2** and **3**, no clear progression of the lower energy bands is observable in the UV/Vis spectra in ethanol. From the solid‐state spectra, a splitting of about 1400 cm^−1^ can be derived, similar to that of **1**. The higher energy bands of solutions of **2** and **3** in ethanol exhibit vibronic progressions as shoulders that are outside the experimentally accessible spectral region of our spectroscopic configuration.

For all acenes, the transitions are shifted, depending on the surrounding medium; the solid‐state spectra are systematically most red‐shifted from the matrix spectra. In contrast to the solid‐state or solution measurements, the absorbance spectra of acenes trapped in solid neon exhibit a clear fine structure, as discussed in more detail the following sections.

The relative energies of the ^1^L_a_ transitions in the solution spectra of substances **1**, **2** and **3** have already been discussed in the literature.^45^ Unsubstituted **1** exhibits the highest value for the first electronic transition of 2.75 eV in the matrix spectrum, followed by four‐fold nitrogen‐substituted derivative **3**, with a first excitation energy of 2.70 eV; both belong to the *D*_2*h*_ point group. The *C*_2*v*_‐symmetric derivative **2** marks an exception in the trend of lowering the energy levels of the ^1^L_a_ transitions upon the introduction of nitrogen atoms into the molecular backbone, with a significantly lower excitation energy of 2.51 eV. This emphasises the impact of symmetry on the optical characteristics of these species. Higher symmetry analytes **1** and **3** may have electronic terms that are Laporte forbidden for excitation from the ground state. However, upon lowering of the symmetry, a mixing of electronic terms is possible, which could explain the bathochromic shift of the first excitation energy of **2**.

The matrix fluorescence spectra of **1** show a complex pattern with a structure that depends on the excitation wavelength with a maximum at *λ*≈535 nm. Compound **2** has the lowest energy emission band, at *λ*=510 nm, with an emission maximum at *λ*=555 nm, and **3** at *λ*=554 nm. The fluorescence data can be found in the Supporting Information.

**Figure 2:**
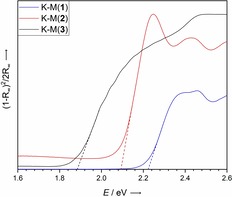
Sections of the Kubelka–Munk plots of acenes **1**–**3** transformed from the reflectance intensities.

The solid‐state diffuse reflectance spectra have a semiconductor‐like shape and have therefore been converted into absorbance spectra (Figure 2) by using the Kubelka–Munk function.^46^ Crossings of the extensions of the edges with the abscissa give estimates for the optical band gaps of 2.22 eV for **1**, 2.09 eV for **2** and 1.88 eV for **3**. The order of the first excitation energies of **1**–**3** in the solid‐state spectra does not correlate with that of the solution or matrix spectra. This is not unexpected, considering the difference in the environment in the solid state, which has a considerable impact on the valence electronic structure.

### Matrix electronic absorbance spectra

#### Tetracene (1)

**Figure 3:**
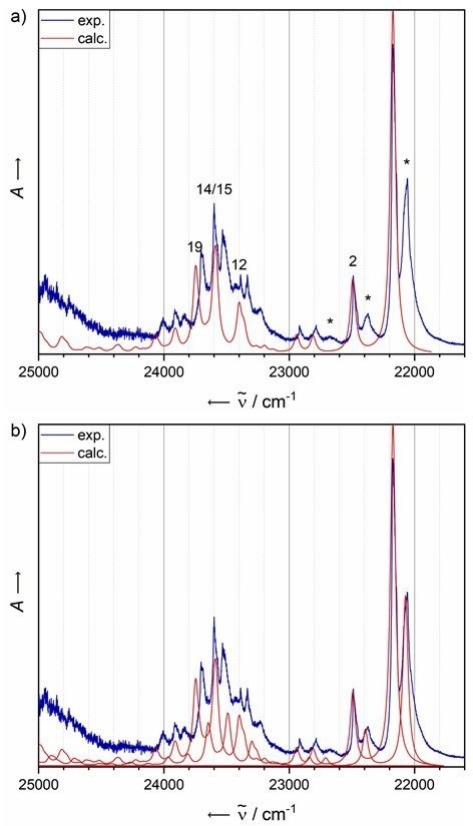
a) A comparison of the experimental (Ne matrix at 4 K after deposition for 5 min at a deposition rate of 0.38 Hz s^−1^ and a neon flow of 3 mL s^−1^) and calculated (B3LYP‐D3BJ/def2‐TZVP) vibrationally resolved electronic spectra of **1**. The computational spectrum was shifted by 1763 cm^−1^ for comparison with that of the experimental spectrum. The most essential vibrational modes are annotated (see the Supporting Information for the full list). Asterisks denote bands assigned to dimers/aggregates. b) Calculated vibrationally resolved electronic spectrum as a superposition of two monomer spectra shifted by about 100 cm^−1^.

**Figure 4:**
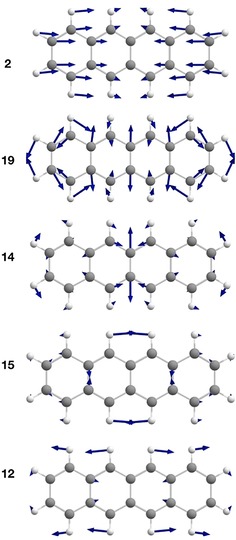
Illustration of the displacement vectors to selected vibrational modes of **1** assigned in the spectrum of Figure 3 a (B3LYP‐D3/def2‐TZVP, threshold: 0.0004).

The most intense signal in the electronic absorbance spectrum of matrix‐isolated **1** lies at 22 173 cm^−1^ and can be assigned to the typical ^1^L_a_ transition of acenes in the Platt nomenclature.^3^ A vibronic progression is clearly visible, with a spacing of 315 and 297 cm^−1^ (exact values of the signals are given in Table 1). The calculated vibrationally resolved electronic spectra reproduce the major signals of vibrational progression stemming from the first excited singlet state (Figures 3 a and 4 and Table 2). Interestingly, a splitting of the signals of 115 cm^−1^ (14.3 meV) is observable and may be attributed to excitonic coupling that comes with the formation of weakly bound, London dispersion driven dimers (J aggregates). Creating a superposition of two monomer spectra, with one shifted by 100 cm^−1^ to lower energies and halved in intensity, results in almost exact reproduction of the experimentally observed spectra (Figure 3 b). Thus, the measured absorbance spectrum in the experiment is generated by the superposition of solely the first excited singlet state (for corresponding attachment and detachment densities, see the Supporting Information).

**Table 1:** Experimental electronic transition energies of **1** in solid Ne matrices at 4 K.

Band system	*ν* [cm^−1^]	*λ* [nm]	*E* [eV]
I	22 173	451	2.75
	22 488	445	2.79
	22 785	439	2.82
I' (dimer)	22 058	453	2.73
	22 373	447	2.77
	22 670	441	2.81
II	22 918	436	2.84

**Table 2:** Most dominant calculated normal modes of **1**, with respect to their coordinate displacement in the first excited singlet state.

Mode	*ν* [cm^−1^]	Dimensionless normal coordinate displacement
2	320	−0.6464
12	1227	−0.4853
14	1411	0.5516
15	1425	−0.5483
19	1575	0.6381

**Figure 5:**
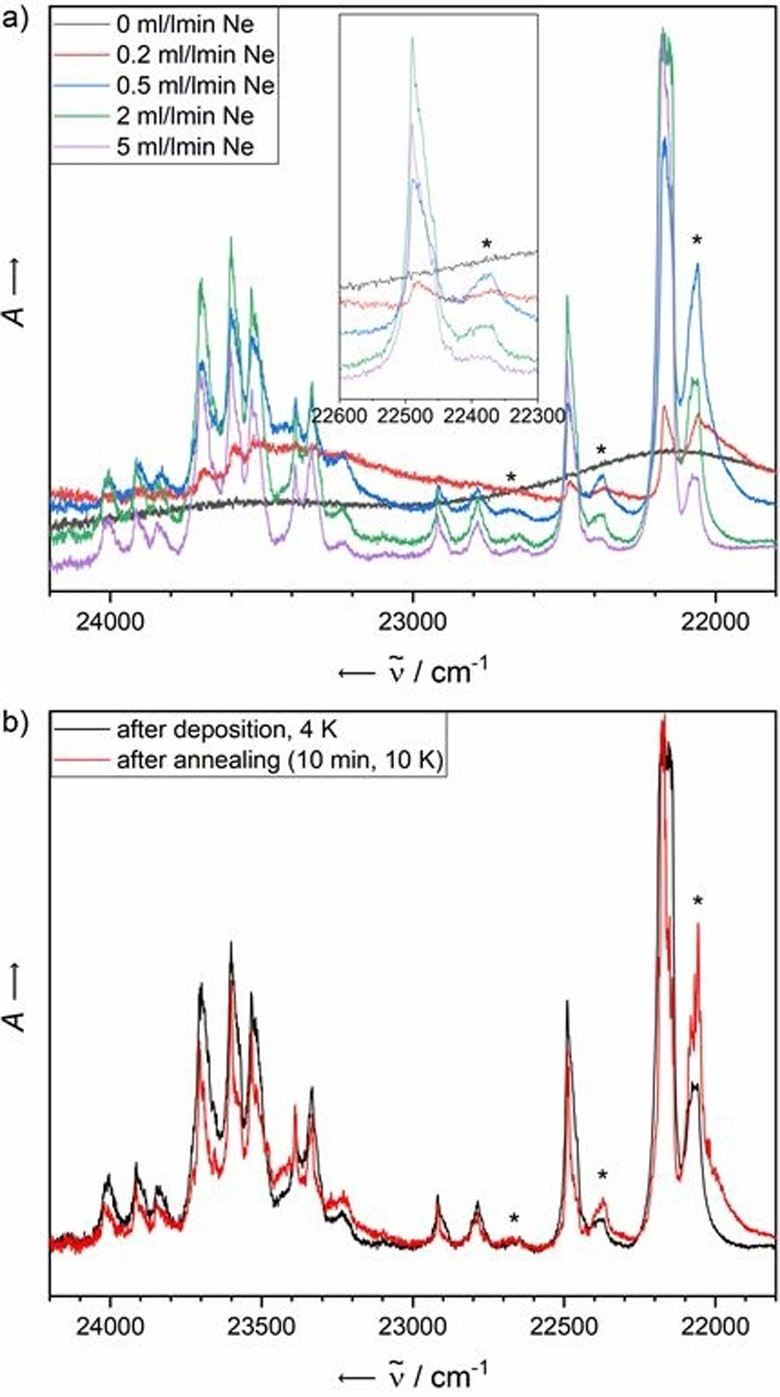
a) Electronic absorbance spectra of **1** in solid Ne recorded at 4 K after deposition for 5 min at a deposition rate of 0.38 Hz s^−1^. The concentration of the analyte has been screened by variation of the Ne flow rate over the range of 0 (leading to an undiluted solid) to 5 mL min^−1^. Asterisks denote bands assigned to dimers/aggregates. b) Electronic absorbance spectra of **1** in solid Ne after deposition for 5 min at a neon flow rate of 3 mL s^−1^ and a deposition rate of 0.38 Hz s^−1^ (black) and after annealing to 10 K for 10 min (red). Both spectra have been recorded at 4 K. Asterisks denote bands assigned to dimers/aggregates.

To further illuminate this observation, we have conducted experiments in which the flow rate of the noble gas has been varied. This is, separate from the applied voltage, one of the parameters that directly affect the final concentration of the matrix, and consequently, the ratio of aggregates to free monomers. The spectra obtained from these experiments are displayed in Figure 5 a, and show a clear dependence of the intensity ratio of the split monomer signals on the substance concentration. Additionally, annealing of matrices is an effective method to support the formation of oligomers that are clearly energetically favoured compared with the statistical, disordered structure obtained upon deposition. Figure 5 b shows the changes in absorbance induced by annealing of the matrix to 10 K for 10 min.

Taking into account identical trends observed in both the Ne flow rate and annealing studies, the splitting of the absorbance signals of **1** in solid Ne can be safely assigned to the dimer. In contrast to matrix isolation studies by Bettinger et al. of the in situ generation of longer acenes by photolysis of α‐diketone precursors,^34^ in which only one additional low‐energy absorption band has been observed, presumably due to geometrical constraints imposed by the matrix material, a splitting of the entire electronic spectrum of **1** was found in our case. Experimental differences in our approach are the concentration range of the dopant and the planar acene **1** being the directly deposited species, which is less susceptible to constraints imposed by the surrounding matrix material as a non‐planar precursor, favouring the formation of dimeric species. Due to the redshifted absorption bands increasing in intensity upon annealing or increasing the concentration, dimer formation is identified as J aggregation.^47^, ^48^ Extensive studies of stacked parallel aromatic dyes, including conformerically confined oligomers linked through a covalent chain and their optical properties,^49^ as well as concentration‐dependent dimer formation in solution,^50^ have been carried out by Würthner et al. and other groups.^49^, ^50^ Compared with one of the first examples of dimeric perylene bisimide (PBI) dyes,^51^ for which a bathochromic shift of 284 cm^−1^ was found after dimerisation, splitting of the absorption bands of **1** (115 cm^−1^) that arises from the co‐existence of mono‐ and dimeric species in the matrix is significantly smaller, but still well resolved, in the spectra. The possibility of recording high‐resolution spectra highlights the advantage of the matrix isolation technique.

It cannot be safely excluded that aggregates larger than dimeric species are formed in the matrix. However, the formation of higher order aggregates should be accompanied by the appearance of additional bands in the spectrum that could not be theoretically reproduced by a simple superposition of two shifted calculated monomer spectra. Additionally, the concentration‐dependent experiments do not reveal changes of the spectra other than relative intensity shifts. Calculations at the B3LYP‐D3BJ/def2‐TZVPP level of theory shed light on the thermodynamic distribution of possible dimer structures and reveal two thermodynamically stable dimers: one in which the tetracene units are parallel, with respect to the long axis of the molecule, but shifted along all three coordinates, and one in which the molecules are additionally rotated by 30° relative to each other. The structures of these two dimer conformations are shown in the Supporting Information. Future theoretical studies will aim at characterising these species in more detail.

#### Benzo[b]phenazine (2)

The visible absorbance spectrum of **2** trapped in a matrix of solid Ne (Figure 7) exhibits its most intense absorption at around 20 880 cm^−1^, which corresponds to a bathochromic shift of 1293 cm^−1^ compared with that of unsubstituted **1**. This band features a vibrational progression, with spacings of 312, 293 and 278 cm^−1^ (see Table 3). Starting at 21 393 cm^−1^ (21 428 cm^−1^), a second band system with a spacing of 240 (205) and 185 cm^−1^ is observable.

**Table 3:** Experimental electronic transition energies of **2** in solid Ne matrices at 4 K.

Band system	*ν* [cm^−1^]	*λ* [nm]	*E* [eV]
additional bands	20 245	494	2.51
	20 369	491	2.53
	20 654	484	2.56
I p (^1^L_a_)	20 880	479	2.59
	21 192	472	2.63
	21 485	465	2.66
	21 763	459	2.70
II	21 393/21 428	467	2.65/2.66
	21 633	462	2.68
	21 818	458	2.71

**Figure 6:**
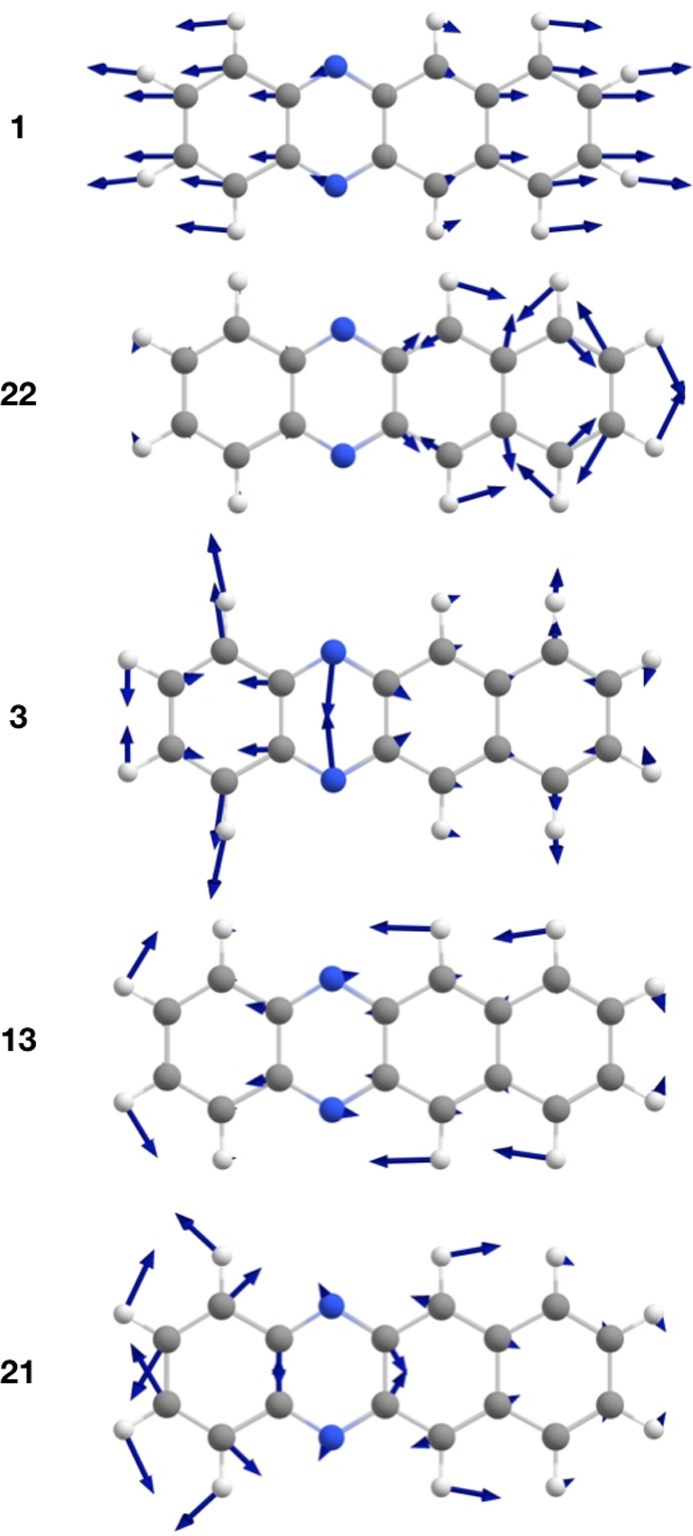
Illustration of the displacement vectors to selected vibrational modes of **2** assigned in the spectrum of Figure7 (B3LYP‐D3/def2‐TZVP, threshold: 0.0004).

**Figure 7:**
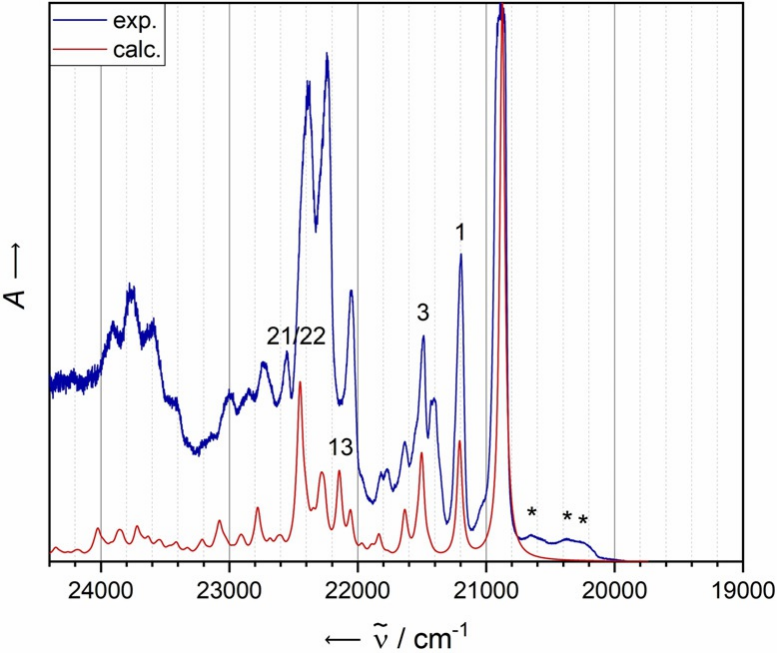
A comparison of the experimental (Ne matrix at 4 K after deposition for 15 min at a deposition rate of 0.15 Hz s^−1^ and a neon flow rate of 3 mL s^−1^) and calculated (B3LYP‐D3BJ/def2‐TZVP) vibrationally resolved electronic spectrum of **2**. The computational spectrum was shifted by 1740 cm^−1^ for comparison with that of the experimental spectrum. The most essential vibrational modes are annotated (see the Supporting Information for the full list). Asterisks denote additional bands with a wavenumber lower than that of the ^1^L_a_ transition.

The calculated monomer spectrum is dominated by the bright S_1_ state (π–π* character; for attachment/detachment densities, see the Supporting Information). The list of contributing normal modes producing the observed vibrational progression through their normal mode coordinate displacement in the first excited state (Figures 6 and 7, and Table 4) can also be found in the Supporting Information. In contrast to **1**, no clear splitting of the signals is observed for **2**. Therefore, aggregation is not observed under the chosen conditions of the matrix experiment. However, there are a few broader signals red‐shifted with respect to the most intense transition (at 20 245, 20 396 and 20 654 cm^−1^). Because, in this case, the calculations suggest the dark n–π* transition to be higher in energy (S_2_) by 2225 cm^−1^, one would assume that the origin of these broad signals would not stem from the aforementioned dark state. This, however, should result in bands within the spectrum that cannot be reproduced by the S_1_ spectrum alone, which is not the case.

**Table 4:** Most dominant calculated normal modes of **2** with respect to their coordinate displacement in the first excited singlet state.

Mode	*ν* [cm^−1^]	Dimensionless normal coordinate displacement
1	333	0.6395
3	628	0.5737
13	1271	0.4921
21	1572	0.4431
22	1578	0.5749

The state ordering is corroborated by high‐level ab initio methods (ADC(2)/def2‐SVPD, see Table S20 in the Supporting Information). The first excited singlet state is the ^1^L_a_ state, followed by an n–π* state. However, one has to keep in mind the approximations made in the simulation, and furthermore effects in the experiment could result in a change of the order of excited states. The case of an energetically lower dark state energetically located beneath the bright state is analysed in detail in the context of compound **3**.

#### Quinoxalino[2,3‐b]quinoxaline (3)

**Figure 8:**
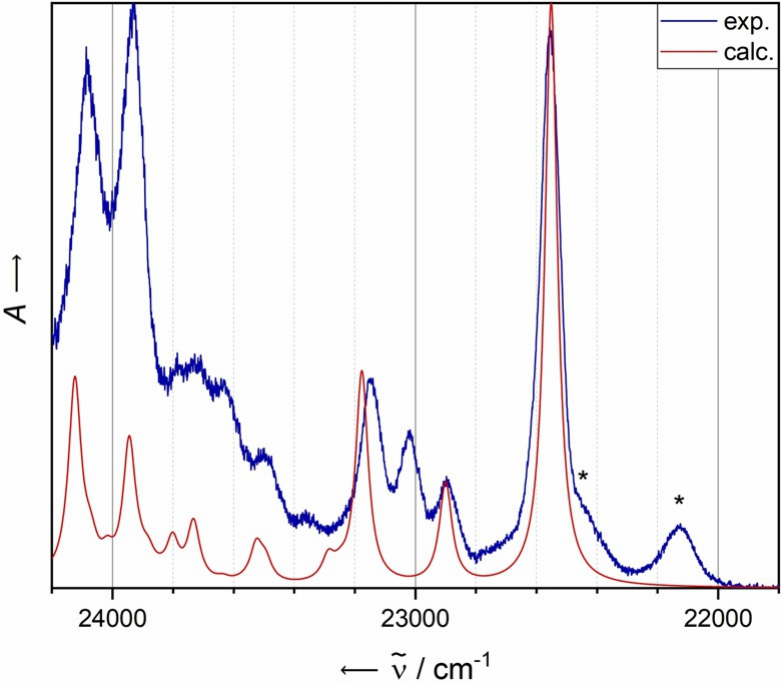
A comparison of the experimental (Ne matrix at 4 K after deposition for 15 min with a deposition rate of 0.68 Hz s^−1^ and a neon flow of 3 mL s^−1^) and calculated (B3LYP‐D3BJ/def2‐TZVP) vibrationally resolved electronic spectra of **3**. The computational spectrum was shifted by 1975 cm^−1^ for comparison with the experimental spectrum. See the Supporting Information for a list of the calculated normal modes. Asterisks denote additional bands with a wavenumber lower than the ^1^L_a_ transition.

Figure 8 shows the visible absorbance spectrum of **3** trapped in a matrix of solid Ne. The most intense transition appears at 22 556 cm^−1^, which corresponds to a hypsochromic shift of 383 cm^−1^ compared with that of unsubstituted **1**. The spectrum exhibits a few lower energy transitions that seem to be more intense than those of the signals in the electronic absorbance spectrum of **2**. In the range of 22 850 to 23 200 cm^−1^, a vibrational progression with increasing intensity is observable that abruptly vanishes. As for the case of **2**, the calculated monomer spectrum does not fully reproduce the experimentally measured spectrum. Even more so, the aforementioned vibrational progression beginning at 22 850 cm^−1^ is not fully reproduced by the calculated monomer spectrum.

The spectrum is dominated by excitation into the S_2_ state (π–π*, see the Supporting Information for the attachment/detachment densities for that excitation), as well as the vibrational progression of the latter. A summary of the bands with the most important intensities in the experimental spectrum is given in Table 5. According to the calculations, the S_1_←S_0_ excitation (n–π*) is lower in energy by 5373 cm^−1^. The high‐level ADC(2)/def2‐SVPD calculations confirm the reversed order of the lowest excited singlet states in the case of **3** relative to that of **2** (see Table S21 in the Supporting Information). The lowest excited singlet state is a dark n–π* state followed by the bright ^1^L_a_ state, producing the experimentally observed electronic absorption spectrum.

**Table 5:** Experimental electronic transition energies of **3** in solid Ne matrices at 4 K.

Band system	*ν* [cm^−1^]	*λ* [nm]	*E* [eV]
additional bands	21 800	495	2.70
	22 125	452	2.74
	22 440	446	2.78
I p (^1^L_a_)	22 556	443	2.80
II	22 897	437	2.84
	23 018	434	2.85
	23 147	432	2.87
III	23 933	418	2.97
	24 087	415	2.99

To investigate the possibility of a Herzberg–Teller vibronic coupling and subsequent intensity borrowing,^52^, ^53^ the Herzberg–Teller approximated vibrationally resolved electronic spectrum for the dark S_1_ state (n–π* character) was calculated and compared with the Franck–Condon approximated spectrum of the bright S_2_ state (see the Supporting Information for the spectra). The two spectra exhibit a spectral overlap, especially in the front part of the S_2_ absorbance spectrum, which could allow for intensity borrowing. Because the red‐shifted peaks appear only for the nitrogen‐substituted species possessing a dark n–π* state, this could further indicate intensity borrowing of dipole‐forbidden transitions due to Herzberg–Teller vibronic coupling.

The question of why no aggregation of **2** and **3** is observed in solid neon matrices cannot be definitely answered yet. Preliminary annealing studies have not offered an answer to this question. Symmetry effects alone are not the driving force for aggregation in the studied series of tetracene derivatives, given that **1** and **3** both belong to the same *D*_2*h*_ point group. Also, the introduction of a dipole in **2** does not seem to result in a different aggregation behaviour in the matrix compared with that of **3**. However, the introduction of nitrogen heteroatoms into the molecular backbone may lead to a reduced spatial extension of the electron density, as well as a reduced polarisability. Dimerisation kinetics and thermodynamics could, in principle, be sensitive to such variations especially in the low‐concentration regime. Additionally, preliminary calculations of dimeric species of the N‐heteroacenes revealed, in both cases, multiple isomers of nearly the same energy, possibly leading to broad bands that may not be distinguished from the background or other signals; thus suggesting an explanation for the absence of sharp dimer bands.

## Conclusion

In this study, a comparative spectroscopic characterisation of a series of unsubstituted and core N‐substituted members of the tetracene family was carried out by using classical solution and solid‐state electronic absorbance spectroscopy, as well as measurements on samples of the species trapped in matrices of solid neon at 4 K. A comparison of the obtained matrix isolation spectra with quantum‐chemical calculations on monomeric molecules emphasises the ability of the matrix isolation technique to closely mimic barely accessible gas‐phase analytics. Compound **1** shows a clear splitting in the matrix electronic absorbance spectrum that is assigned to the formation of dimeric species (J aggregates) upon deposition. This assignment is supported by concentration‐dependent studies and annealing experiments, which reveal an increased proportion of aggregation in matrices of higher concentration of **1** or after annealing. The possibility of experimentally measuring such splitting with a high resolution allows for the precise analysis of the strength/thermodynamics of dimer interactions (e.g., exciton coupling) in combination with computational simulations. Furthermore, the N‐substituted molecules exhibit different behaviour in the matrix. The introduction of nitrogen atoms into the molecular backbone gives rise to dark n–π* excited states, namely, the S_1_ state of **3**. The possibility of intensity borrowing arises and can explain the observation of additional bands in the spectrum that are red‐shifted with respect to the strong π–π* transition.

## Experimental Section

Compound **1** was purchased from TCI Europe and purified by re‐sublimation. Compounds **2** and **3** were synthesised according to a literature procedure and purified by means of column chromatography on aluminium oxide.^54^ Additionally, compound **2** was purified by recrystallisation from chloroform. Matrix isolation experiments were performed according to standard techniques in this form, which were first reported by Pimentel et al.^41^ and thereafter tremendously developed by Andrews et al.^55^, ^56^, ^57^, ^58^ Details of the matrix setup in Heidelberg can be found elsewhere.^59^ The substances were evaporated in a water‐cooled Knudsen‐type effusion cell containing a graphite tube inside a ceramic unit surrounded by a Ta heating coil. The deposition rate was determined through preliminary reference calibration measurements by using a separate quartz apparatus (see the Supporting Information for details). Matrices were created by co‐deposition of the substances and Ne (Air Liquide, 99.999%) on a Rh‐coated Cu surface cooled to 4.2 K by using a pulse‐tube cooler (Vericold) and a closed‐cycle helium cryostat. During deposition, the flow of the gas was kept constant by using a flow controller (EL‐FLOW, Bronkhorst). Visible and IR spectra were obtained with a Bruker Vertex 80v spectrometer at resolutions of 1 and 0.1 cm^−1^, respectively, with a Globar source, a KBr beam splitter and a mercury cadmium telluride (MCT) detector for the mid‐IR range and a tungsten lamp, a CaF_2_ beam splitter and a Si diode detector for the visible range. Fluorescence spectra were recorded with a Symphony II charge‐coupled device (CCD) detector (Horiba) by using a binning factor of one after excitation with an Ar laser (Innova 90c‐A3; Coherent). To simulate the vibrationally resolved electronic spectra, a time‐dependent approach (independent mode displaced harmonic oscillator model with temperature effects) derived from the time‐dependent wave packet theory derived by Heller was used, as implemented in the ORCA 3.03 software package (see the Supporting Information for computational details).^60^, ^61^ To calculate the Herzberg–Teller approximated absorbance spectra, the time‐independent approximation implemented within the Gaussian 16 (Rev. B.01) program was used.^62^, ^63^ The attachment and detachment densities for the investigated transitions were calculated by using the Q‐Chem 5.1 software package.^64^

### Determination of deposition rate calibration curves

**Figure 9:**
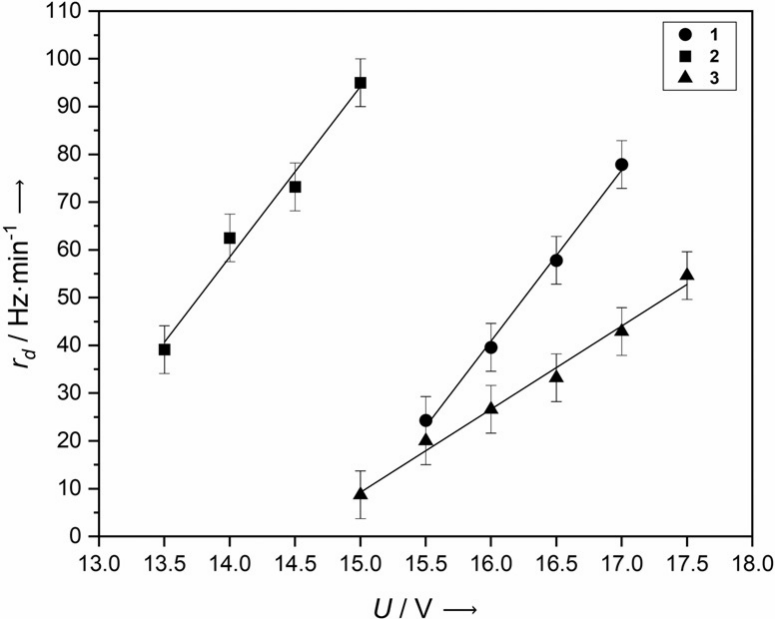
Dependence of the deposition rate, *r*_d_, on the applied voltage for **1**–**3**.

For an accurate discussion of electronic absorbance spectra of matrix isolated analytes, it is important to quantify the amount of deposited material. Therefore, we conducted calibration measurements of all analysed species by using an oscillating quartz apparatus (see the Supporting Information for more technical details). These measurements give a linear dependence of the deposition rates of **1**–**3** on the applied voltage over the studied ranges. Figure 9 shows the calibration curves of **1**–**3**.

Although **1** and **2** had different vapour pressures, the curves exhibited similar slopes of 35.8 and 35.7 Hz min^−1^ V^−1^, respectively. Four‐fold nitrogen‐substituted **3** had a smaller slope of 17.4 Hz min^−1^ V^−1^.

## Conflict of interest

The authors declare no conflict of interest.

## Supporting information

As a service to our authors and readers, this journal provides supporting information supplied by the authors. Such materials are peer reviewed and may be re‐organized for online delivery, but are not copy‐edited or typeset. Technical support issues arising from supporting information (other than missing files) should be addressed to the authors.

Supplementary
