# Supplementary material for: Low‐Energy Electronic Excitations of N‐Substituted Heteroacene Molecules: Matrix Isolation Spectroscopy in Concert with Quantum‐Chemical Calculations
Source: Chemistry. 2019 Oct 30;25(66):15147–54. doi: 10.1002/chem.201903371 (PMC6899788; doi:10.1002/chem.201903371)
Supplement: Supplementary file 1 — Supplementary [file CHEM-25-15147-s001.pdf]

CHEMISTRY
A European Journal

Supporting Information

Low-Energy Electronic Excitations of N-Substituted Heteroacene Molecules: Matrix Isolation Spectroscopy in Concert with Quantum-Chemical Calculations

Jean Thusek,[a] Marvin Hoffmann,[b] Olaf Hübner,[a] Olena Tverskoy,[c] Uwe H. F. Bunz,*[c] Andreas Dreuw,*[b] and Hans-Jörg Himmel*[a]

chem_201903371_sm_miscellaneous_information.pdf

Table of contents

1. Technical details 2
   1.1. Matrix creation 2
   1.2. Quartz microbalance setup 3
2. Fluorescence spectra 4
   2.1. Tetracene (**1**) 4
   2.2. Benzo[*b*]phenazine (**2**) 5
   2.3. Quinoxalino[2,3-*b*]quinoxaline (**3**) 5
3. Structures 6
   3.1. Cartesian coordinates (in Å) for tetracene (**1**) 6
   3.2. Cartesian coordinates (in Å) for benzo[*b*]phenazine (**2**) 7
   3.3. Cartesian coordinates (in Å) for quinoxalino[2,3-*b*]quinoxaline (**3**) 8
   3.4. Cartesian coordinates (in Å) for quinoxalino[2,3-*b*]quinoxaline (**3**) – S1 Minimum 9
   3.5. Cartesian coordinates (in Å) for quinoxalino[2,3-*b*]quinoxaline (**3**) – S2 Minimum 10
   3.6. Cartesian coordinates for the tetracene (**1**) dimer 1 11
   3.7. Cartesian coordinates for the tetracene (**1**) dimer 2 13
4. Vibrationally-Resolved Spectra Contributions (IMDHOT) 15
   4.1. Tetracene (**1**) 15
   4.2. Benzo[*b*]phenazine (**2**) 18
   4.3. Quinoxalino[2,3-*b*]quinoxaline (**3**) 21
5. High-level calculations of the lowest excited singlet states 24
   5.1. Tetracene (**1**) 24
   5.2. Benzo[*b*]phenazine (**2**) 24
   5.3. Quinoxalino[2,3-*b*]quinoxaline (**3**) 24

1. Technical details

1.1. Matrix creation

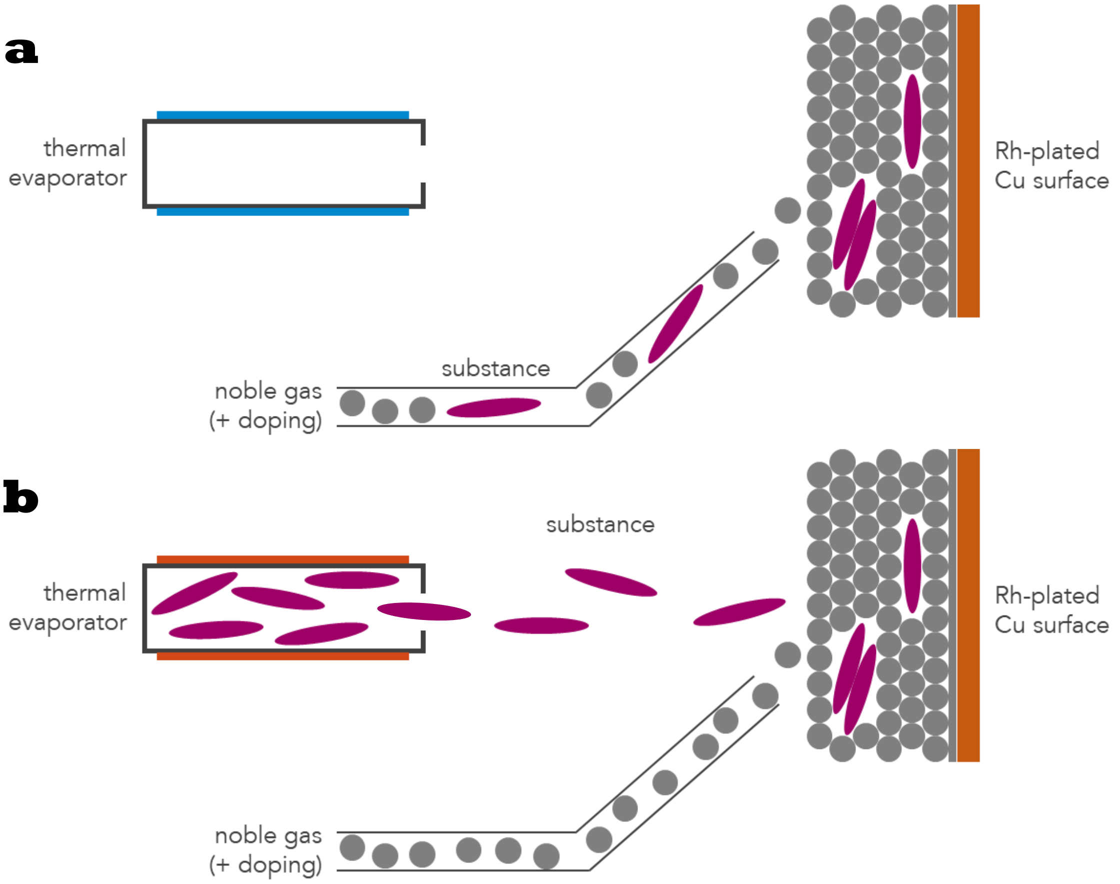

Figure S1: Schematic representation of possible evaporation configurations for organic substances. The Rh surface plated on a Cu block is cooled to 4 K using a closed-cycle He cryostat. **a** Liquids or substances with a high vapor pressure can be mixed with the noble gas prior to deposition in a defined concentration using a baratron to monitor the partial pressure. **b** Solids such as the investigated acenes are heated in a Knudsen-type effusion cell: A graphite tube with an aperture of 0,5 mm containing 5 mg of the respective substance is inserted in a ceramic oven that is surrounded by a Ta coil to which voltage is applied. The substances are trapped by codeposition with the noble gas on the Rh surface.

1.2. **Quartz microbalance setup**

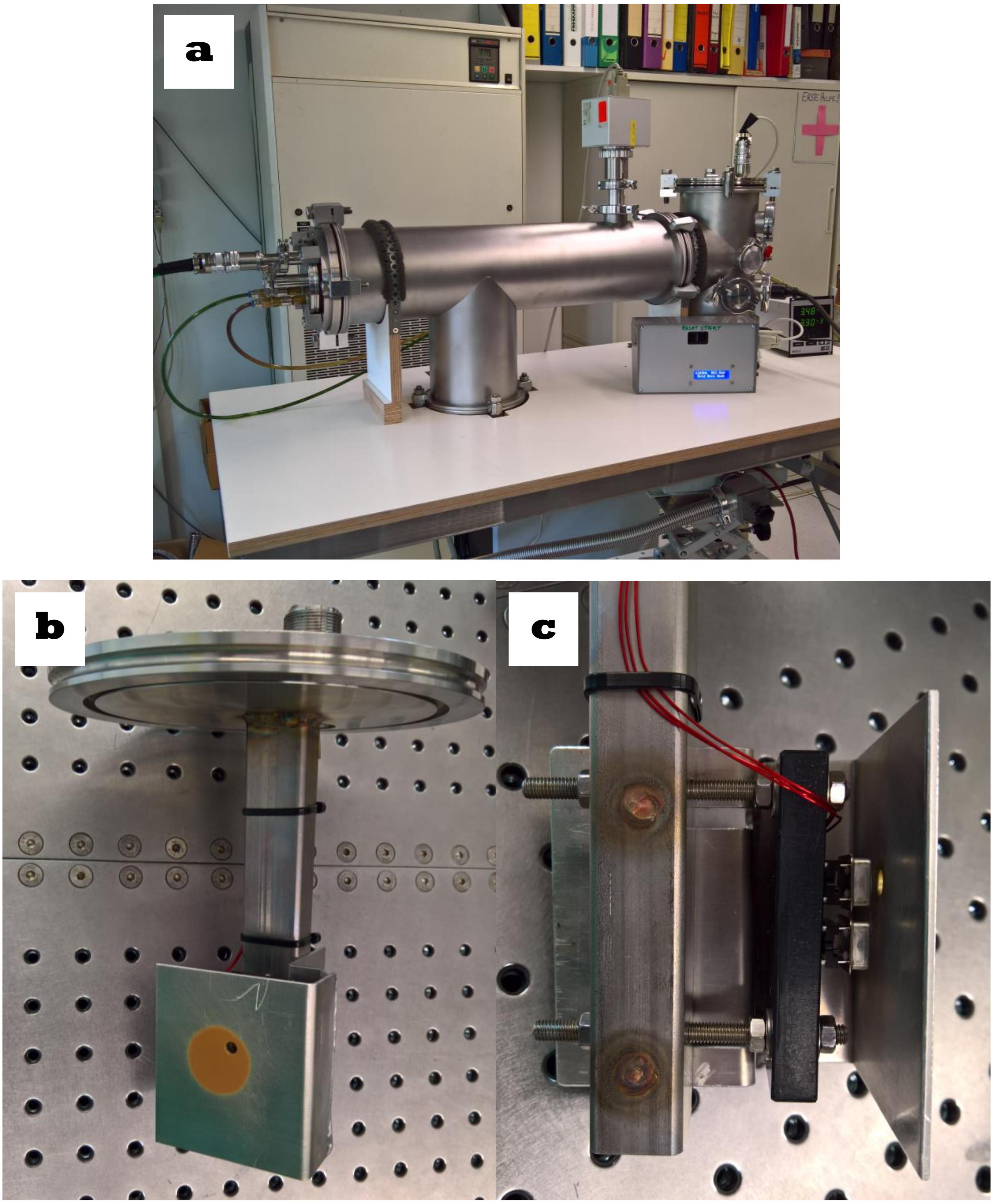

Figure S2: Quartz microbalance setup for the determination of deposition rates. **a** Overview of the setup. The evaporator is inserted in the left part of the chamber, the right part contains the quartz microbalance device. **b** Inset containing the quartz microbalance shielded by a plate with an aperture in front of the deposition quartz. The quartz-evaporator distance is the same as in the matrix setup (39 mm). **c** Side view of the inset showing the deposition quartz behind the aperture (upper quartz) and the reference quartz shielded by the plate (lower quartz).

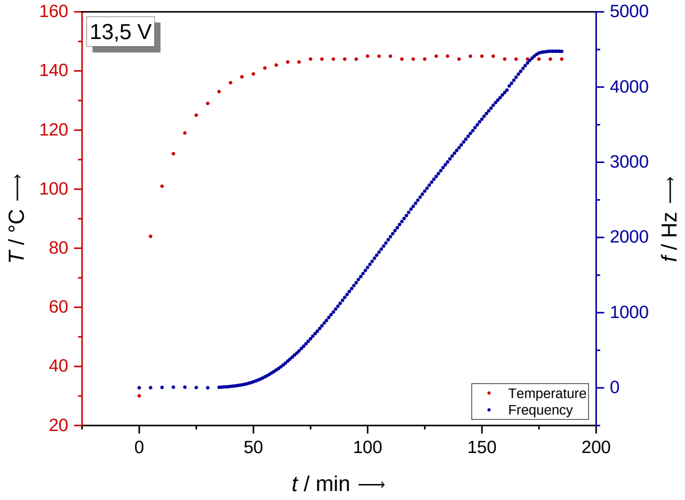

Figure S3: Exemplary deposition curve of benzo[*b*]phenazine (**2**) evaporated at a voltage of 13,5 V leading to one point of the calibration curves shown in figure 9 (main text) as the slope of the linear part of the frequency function.

2. Fluorescence spectra

2.1. Tetracene (1)

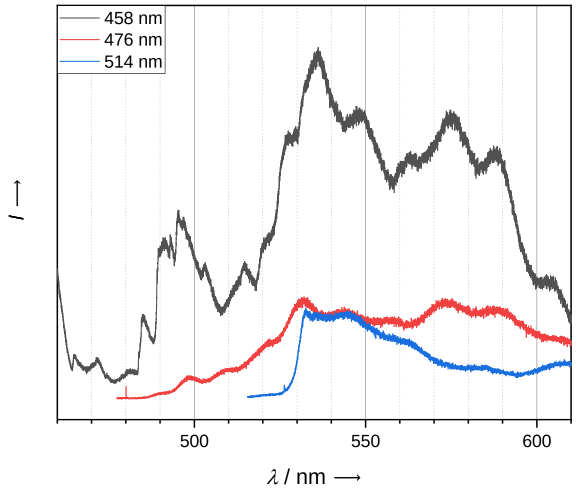

Figure S4: Fluorescence spectra of tetracene (**1**) in solid Ne at 4 K after deposition for 5 min with a deposition rate of 0.38 Hz·s^{-1} and a neon flow of 0.5 ml·s^{-1} recorded with different excitation wavelengths (458 nm at 20 mW, 476 nm and 514 nm at 200 mW).

2.2. **Benzo[*b*]phenazine (2)**

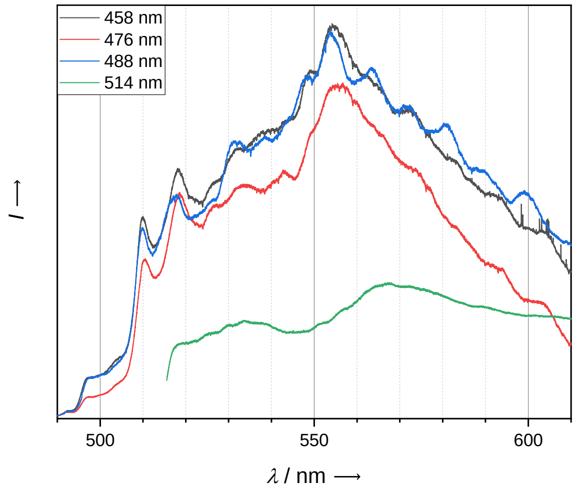

Figure S5: Fluorescence spectra of benzo[*b*]phenazine (**2**) in solid Ne at 4 K after deposition for 5 min with a deposition rate of 0.68 Hz·s^{-1} and a neon flow of 3 ml·s^{-1} recorded with different excitation wavelengths (488 nm at 5 mW, 476 nm and 514 nm at 20 mW).

2.3. **Quinoxalino[2,3-*b*]quinoxaline (3)**

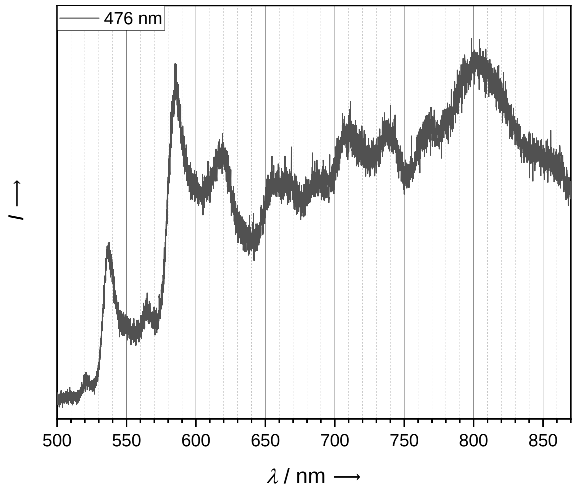

Figure S6: Fluorescence spectrum of quinoxalino[2,3-*b*]quinoxaline (**3**) in solid Ne at 4 K after deposition for 15 min with a deposition rate of 0.88 Hz·s^{-1} and a neon flow of 3 ml·s^{-1} recorded with an excitation wavelength of 476nm (200 mW).

3. Structures

3.1. Cartesian coordinates (in Å) for tetracene (1)

C	2.599158	1.407421	-0.000020
C	2.536587	-0.037839	-0.000004
C	1.298419	-0.661005	-0.000019
C	0.099214	0.071942	-0.000025
C	1.420843	2.139270	-0.000036
C	0.162003	1.517489	-0.000025
C	-1.159621	-0.549844	-0.000033
C	-2.337943	0.182003	-0.000016
C	-1.037203	2.250435	-0.000022
C	-2.275372	1.627266	-0.000003
C	-3.622334	-0.442745	-0.000012
C	-4.763358	0.298588	-0.000003
C	-4.702031	1.723700	-0.000005
C	-3.501320	2.363084	0.000011
C	3.883540	2.032178	-0.000009
C	3.762535	-0.773654	0.000021
H	1.249052	-1.744569	-0.000007
H	1.470465	3.222777	-0.000035
H	-1.209230	-1.633348	-0.000033
H	-0.987835	3.334001	-0.000014
H	-3.668849	-1.525597	-0.000009
H	-5.729129	-0.190384	-0.000008
H	-5.621632	2.294747	0.000020
H	-3.452850	3.445881	0.000021
C	4.963251	-0.134266	0.000017
C	5.024559	1.290847	0.000014
H	3.930049	3.115030	-0.000016
H	3.714068	-1.856451	0.000028
H	5.882854	-0.705309	0.000050
H	5.990328	1.779822	0.000022

3.2. **Cartesian coordinates (in Å) for benzo[*b*]phenazine (2)**

C	-4.765952	-0.714080	-0.000918
C	-4.766268	0.715157	-0.000101
C	-3.599796	1.414500	0.000362
C	-2.348021	0.726622	0.000009
C	-2.347701	-0.724436	-0.000894
C	-3.599164	-1.412893	-0.001321
N	-1.217557	1.419737	0.000511
C	-0.063391	0.725277	0.000078
C	-0.063072	-0.722045	-0.000838
N	-1.216929	-1.417035	-0.001332
C	1.159888	1.411493	0.000517
C	2.365920	0.726814	0.000100
C	2.366232	-0.722484	-0.000748
C	1.160505	-1.407714	-0.001217
C	3.622488	1.407965	0.000510
C	4.793480	0.716002	0.000136
C	4.793799	-0.710623	-0.000656
C	3.623095	-1.403086	-0.001092
H	-5.712046	-1.240117	-0.001252
H	-5.712600	1.240767	0.000186
H	-3.575825	2.496495	0.001038
H	-3.574697	-2.494880	-0.001989
H	3.620546	2.491413	0.001164
H	5.737197	1.246114	0.000467
H	5.737745	-1.240326	-0.000935
H	3.621603	-2.486535	-0.001736
H	1.134986	2.494268	0.001219
H	1.136094	-2.490500	-0.001918

3.3. **Cartesian coordinates (in Å) for quinoxalino[2,3-*b*]quinoxaline (3)**

C	-4.695163	-0.714401	-0.000894
C	-4.695477	0.715537	-0.000116
C	-3.530578	1.416661	0.000345
C	-2.279186	0.727919	0.000013
C	-2.278868	-0.725710	-0.000909
C	-3.529955	-1.415011	-0.001300
N	-1.150350	1.422151	0.000553
C	-0.001834	0.726799	0.000106
C	-0.001517	-0.723573	-0.000911
N	-1.149730	-1.419439	-0.001409
N	1.146378	1.422666	0.000611
C	2.275519	0.728941	0.000123
C	2.275834	-0.724688	-0.000807
N	1.147002	-1.418924	-0.001359
C	3.526601	1.418254	0.000530
C	4.691809	0.717649	0.000127
C	4.692131	-0.712288	-0.000657
C	3.527234	-1.413417	-0.001133
H	-5.641865	-1.239178	-0.001199
H	-5.642414	1.239891	0.000144
H	-3.505873	2.498342	0.000997
H	-3.504768	-2.496682	-0.001948
H	3.501411	2.499924	0.001190
H	5.638511	1.242429	0.000447
H	5.639068	-1.236643	-0.000908
H	3.502551	-2.495099	-0.001786

3.4. **Cartesian coordinates (in Å) for quinoxalino[2,3-*b*]quinoxaline (3) – S1 Minimum**

Excitation energy = 1.0918 eV

C	-4.742573	-0.704238	-0.000000
C	-4.742573	0.704238	-0.000000
C	-3.559969	1.406341	-0.000000
C	-2.326907	0.723011	-0.000000
C	-2.326907	-0.723011	-0.000000
C	-3.559969	-1.406341	-0.000000
N	-1.163553	1.381405	0.000000
C	0.000000	0.780183	0.000000
C	-0.000000	-0.780183	0.000000
N	-1.163553	-1.381405	-0.000000
N	1.163553	1.381405	0.000000
C	2.326907	0.723011	0.000000
C	2.326907	-0.723011	0.000000
N	1.163553	-1.381405	0.000000
C	3.559969	1.406341	0.000000
C	4.742573	0.704238	0.000000
C	4.742573	-0.704238	0.000000
C	3.559969	-1.406341	0.000000
H	-5.683337	-1.239077	-0.000000
H	-5.683337	1.239077	-0.000000
H	-3.541518	2.487551	-0.000000
H	-3.541518	-2.487551	-0.000000
H	3.541518	2.487551	0.000000
H	5.683337	1.239077	0.000000
H	5.683337	-1.239077	0.000000
H	3.541518	-2.487551	0.000000

3.5. **Cartesian coordinates (in Å) for quinoxalino[2,3-*b*]quinoxaline (3) – S2 Minimum**

Excitation energy = 2.5354 eV

C	-4.730211	-0.697515	0.000000
C	-4.730211	0.697515	0.000000
C	-3.525295	1.397030	0.000000
C	-2.288131	0.716436	0.000000
C	-2.288131	-0.716436	0.000000
C	-3.525295	-1.397030	0.000000
N	-1.145073	1.427813	0.000000
C	0.000000	0.734900	0.000000
C	-0.000000	-0.734900	0.000000
N	-1.145073	-1.427813	0.000000
N	1.145073	1.427813	-0.000000
C	2.288131	0.716436	-0.000000
C	2.288131	-0.716436	-0.000000
N	1.145073	-1.427813	-0.000000
C	3.525295	1.397030	-0.000000
C	4.730211	0.697515	-0.000000
C	4.730211	-0.697515	-0.000000
C	3.525295	-1.397030	-0.000000
H	-5.666231	-1.240259	0.000000
H	-5.666231	1.240259	0.000000
H	-3.503917	2.479215	0.000000
H	-3.503917	-2.479215	0.000000
H	3.503917	2.479215	-0.000000
H	5.666231	1.240259	-0.000000
H	5.666231	-1.240259	-0.000000
H	3.503917	-2.479215	-0.000000

3.6. **Cartesian coordinates for the tetracene (1) dimer 1**

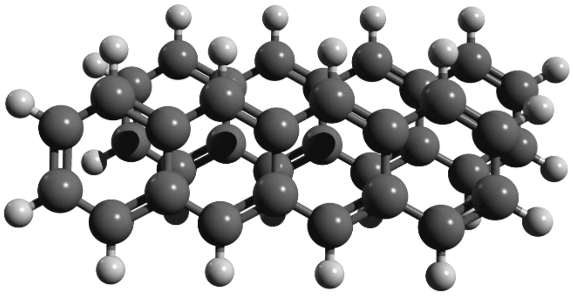

Figure S7: Structure of the parallel dimer conformer of tetracene (**1**).

C	7.0618712486	1.4428259178	-0.0412230321
C	7.0419761284	-0.0028790687	-0.0482295398
C	5.8246830536	-0.6635857366	-0.0990189004
C	4.6058725501	0.0310848732	-0.1571023559
C	5.8637508851	2.1380230211	-0.0963800254
C	4.6257180202	1.4772441101	-0.1586722420
C	3.3681292655	-0.6300449592	-0.2086964494
C	2.1698726632	0.0632466147	-0.2732210025
C	3.4062099318	2.1712514974	-0.2201609124
C	2.1897198476	1.5087967559	-0.2801319780
C	0.9062288006	-0.6012527725	-0.3233603193
C	-0.2552826099	0.1028123856	-0.3857346026
C	-0.2353530142	1.5281060163	-0.3973918173
C	0.9434509621	2.2050737994	-0.3449766723
H	5.8091636304	-1.7470254188	-0.0872590956
H	5.8792263421	3.2223114058	-0.0925933734
H	3.3527639665	-1.7133905423	-0.1904473350
H	3.4200698876	3.2555836172	-0.2225993786
H	0.8928446341	-1.6837568405	-0.2977203377
H	-1.2050427936	-0.4147148968	-0.4066784346
H	-1.1720940339	2.0691700816	-0.4386441691
H	0.9578196073	3.2886636039	-0.3495492141
C	8.3261823245	2.1050052448	0.0271279472
C	9.4871148662	1.3967543737	0.0799765015
C	9.4673734554	-0.0292437410	0.0653957987
C	8.2862051147	-0.7016690914	0.0053337165
H	8.3411409037	3.1885113337	0.0356814329
H	10.4374722681	1.9133269034	0.1306595340
H	10.4025638797	-0.5732032250	0.1078314260
H	8.2675452100	-1.7845009192	0.0064399992

C	1.9077341727	-1.7464354354	3.0963426376
C	0.7097184516	-1.0510471049	3.0412692970
C	-1.6955400136	0.4214095035	2.9347842212
C	1.9472670793	1.0551595963	3.0989919449
C	0.7298616540	0.3946514746	3.0482727440
C	-0.5142617468	1.0936453738	2.9947961565
C	-0.5546935474	-1.7130244137	2.9729512303
C	-1.7155160490	-1.0045837164	2.9201586972
C	3.1659639824	0.3602898396	3.1570643126
C	4.4038205458	1.0212074414	3.2086541053
C	4.3652768229	-1.7800806085	3.2200282925
C	5.6019650362	0.3277168464	3.2731613462
C	5.5818758237	-1.1178313286	3.2799891398
C	3.1458788034	-1.0858682975	3.1586016582
C	6.8280349990	-1.8143176114	3.3448186216
C	6.8657139717	0.9920147507	3.3233507063
C	8.0069432758	-1.1375352036	3.3973026197
C	8.0271053284	0.2877549786	3.3857245502
H	4.3512024297	-2.8644103872	3.2223457345
H	6.8792474338	2.0745158192	3.2976999152
H	-2.6306488945	0.9655202058	2.8924742760
H	8.9769514272	0.8051256226	3.4066670189
H	6.8135003254	-2.8979050959	3.3493730832
H	8.9435951590	-1.6787550319	3.4385448228
H	-0.4954318269	2.1764747762	2.9937763064
H	4.4193744356	2.1045517358	3.1904658916
H	1.9629356874	2.1385975833	3.0872673117
H	1.8920828488	-2.8307215717	3.0925231397
H	-0.5698460683	-2.7965270062	2.9644181790
H	-2.6659572871	-1.5210061981	2.8695268714

3.7. **Cartesian coordinates for the tetracene (1) dimer 2**

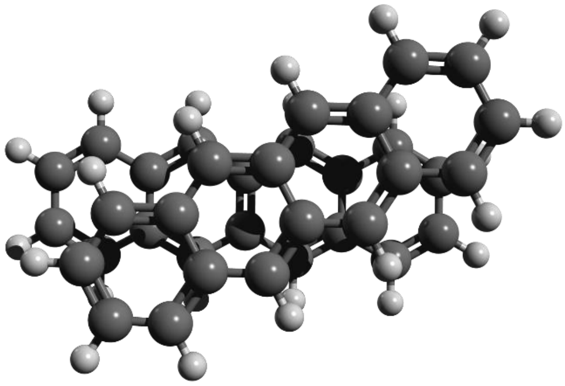

Figure S8: Structure of the rotated dimer conformer of tetracene (**2**).

C	2.4446964263	0.4543332807	0.3146570742
C	2.4426617641	-0.9907822323	0.2635688789
C	1.2153667827	-1.6639518718	0.1631962915
C	0.0083336606	-0.9831119778	0.1173568149
C	0.0075327450	0.4597846850	0.1937335663
C	1.2159981346	1.1343941970	0.2882778431
C	-1.2482499000	1.1398131392	0.1770534933
C	-2.4191873989	0.4514093042	0.0749100757
C	-2.4162146220	-0.9706500647	-0.0172269381
C	-1.2432592589	-1.6609597372	0.0077682661
H	-1.2499369040	2.2216288397	0.2416655411
H	-3.3626873069	0.9824659564	0.0597282615
H	-3.3576955334	-1.4989326433	-0.1003306138
H	-1.2385174411	-2.7426746230	-0.0468923481
C	3.6727596518	1.1272797901	0.4097210491
C	4.8792965914	0.4448785540	0.4618346009
C	4.8760463884	-0.9998656425	0.4296249013
C	3.6682526818	-1.6736747466	0.3312375511
C	6.1315108815	1.1224655459	0.5609357025
C	7.3006499461	0.4295291861	0.6287802508
C	7.2986101845	-0.9947810910	0.6088132856
C	6.1273223971	-1.6832413453	0.5151727151
H	6.1316296495	2.2046062677	0.5967228819
H	8.2406181194	0.9579385443	0.7162118795
H	8.2391942292	-1.5275271340	0.6718545744
H	6.1248121749	-2.7668533173	0.4991843438
C	-0.5645921706	-2.9703100870	3.3696519165
C	5.2027388438	1.7182678290	3.8192151856

C	5.9951771722	2.9061109244	3.8451166726
C	0.9020705254	-0.5688478688	3.5384553566
C	-1.2320271708	-1.7118336821	3.3648128096
C	0.7928465177	-3.0327565085	3.4597300452
C	-0.5218590233	-0.5537640648	3.4429803862
C	5.8764149019	0.4416187000	3.8826369647
C	1.5800855440	-1.8449064730	3.5562027501
C	3.7275863598	-0.7014727874	3.7297213738
C	3.0504343683	0.5756371155	3.6920393148
C	8.0143670629	1.5844760153	4.0236106716
C	7.3522580058	2.8438721282	3.9442588207
C	7.2998878943	0.4262838207	3.9891303328
C	1.6500777334	0.5976154991	3.6003043533
C	5.1273316858	-0.7239531479	3.8275313573
C	3.8189406469	1.7507587780	3.7277473951
C	2.9629196654	-1.8772152628	3.6517103279
H	-1.1474191724	-3.8800864279	3.2980726015
H	7.8014088430	-0.5326488050	4.0344718106
H	9.0937362554	1.5566909074	4.1045053407
H	-1.0274596661	0.4033489761	3.4184067828
H	7.9382655950	3.7541125739	3.9666395708
H	5.4908741589	3.8637890678	3.7899932682
H	-2.3101738735	-1.6828891409	3.2803080839
H	1.3013705437	-3.9897644360	3.4644024209
H	1.1409287160	1.5537420466	3.5699597060
H	3.4724794521	-2.8339406208	3.6632303636
H	5.6358256267	-1.6804791714	3.8522754770
H	3.3125133231	2.7084751516	3.6836901552
H	1.2127677894	-2.7469770103	0.1287750041
H	3.6647457710	-2.7574593561	0.3090971644
H	3.6761751430	2.2101891202	0.4505979668
H	1.2162008879	2.2173333333	0.3421583065

4. Vibrationally-Resolved Spectra Contributions (IMDHOT)

4.1. **Tetracene (1)**

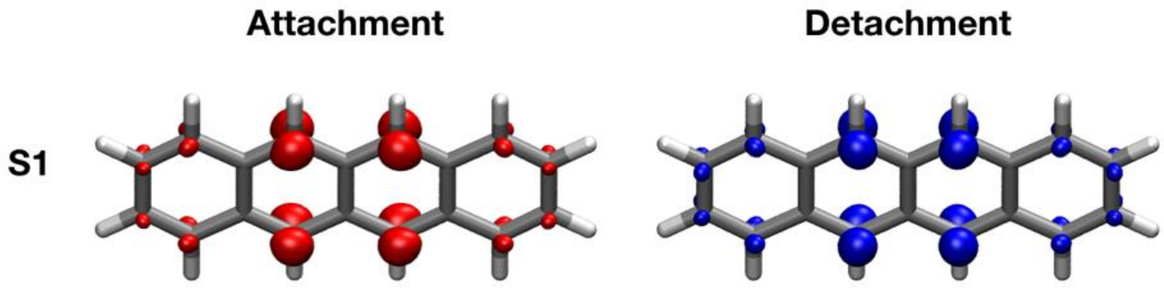

Figure S9: Attachment and detachment densities of the first excited singlet state of **1** (B3LYP/def2-TZVP). The plotted surfaces correspond to a surface of 0.005 e·Bohr^{-3}.

Mode	Frequency [cm⁻¹]	Dimensionless Normal Coordinate Displacement
1	304	0.0540
2	320	-0.6464
3	506	0.0264
4	634	0.2241
5	768	-0.3166
6	790	-0.0102
7	872	-0.0595
8	1024	-0.1773
9	1153	-0.0179
10	1188	0.3280
11	1206	-0.0256
12	1227	-0.4853
13	1364	0.0094
14	1411	0.5516
15	1425	-0.5483
16	1484	-0.1802
17	1486	0.0624
18	1559	-0.1717
19	1575	0.6381
20	1648	0.0159
21	1663	-0.0092
22	3156	-0.0004
23	3191	0.0174

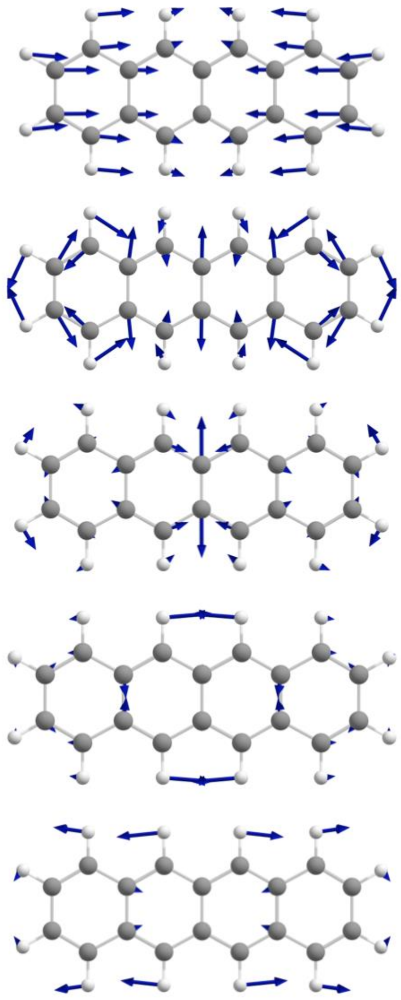

Figure S10: Normal modes and their corresponding displacement vectors with respect to the first excited state above a threshold of 0.0004 to the vibronic spectrum of **1**.

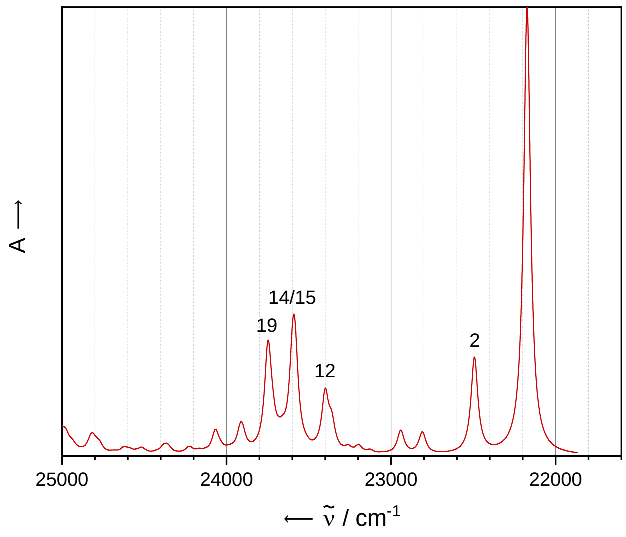

Figure S11: Calculated vibrationally-resolved electronic spectrum of **1** (shifted by 1763 cm^{-1}) with the most essential vibrational modes annotated (B3LYP-D3/def2-TZVP).

4.2. **Benzo[*b*]phenazine (2)**

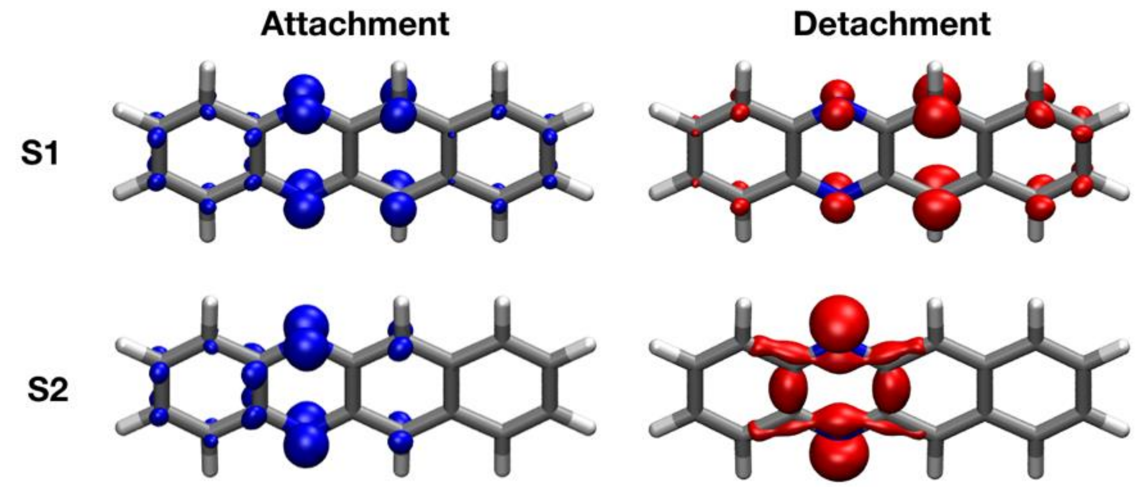

Figure S12: Attachment and detachment densities of the first two excited singlet states of **2** (B3LYP/def2-TZVP). The plotted surfaces correspond to a surface of 0.005 e·Bohr^{-3}.

Mode	Frequency [cm-1]	Dimensionless Normal Coordinate Displacement
1	333	0.6395
2	568	0.1317
3	628	0.5737
4	634	0.1338
5	747	0.1424
6	762	0.3755
7	891	0.0878
8	1014	0.0899
9	1022	0.1423
10	1146	0.0296
11	1182	0.3652
12	1191	0.0315
13	1271	0.4921
14	1333	0.0272
15	1386	0.3455
16	1410	0.3443
17	1428	0.2888
18	1473	0.2617
19	1503	0.0572
20	1536	0.2788
21	1572	0.4431
22	1578	0.5749
23	3180	0.0097
24	3182	0.0038
25	3193	0.0165
26	3198	0.0045

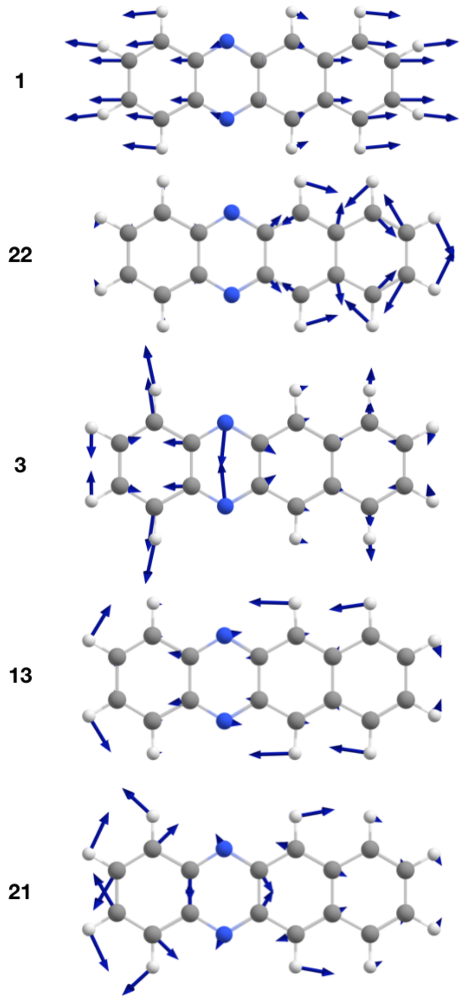

Figure S13: Normal modes and their corresponding displacement vectors with respect to the first excited state above a threshold of 0.0004 to the vibronic spectrum of **2**.

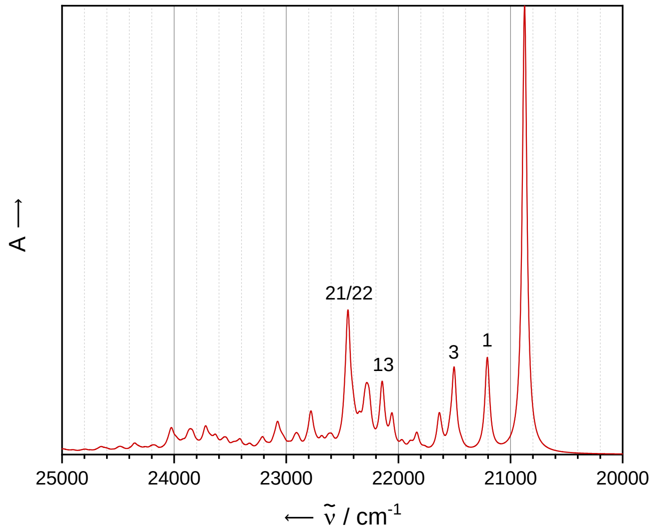

Figure S14: Calculated vibrationally-resolved electronic spectrum of **2** (shifted by 1740 cm^{-1}) with the most essential vibrational modes annotated (B3LYP-D3/def2-TZVP).

4.3. **Quinoxalino[2,3-*b*]quinoxaline (3)**

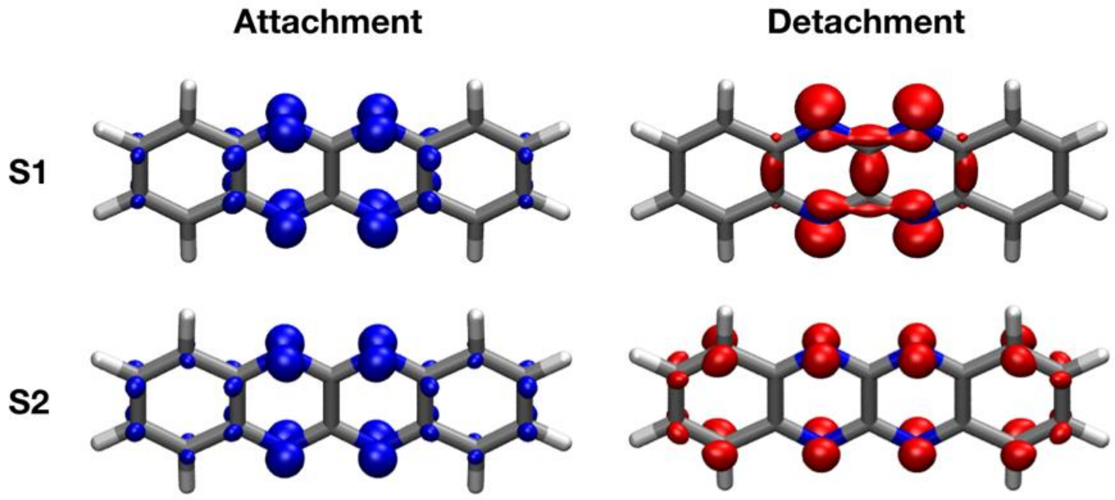

Figure S15: Attachment and detachment densities of the first two excited singlet states of **3** (B3LYP/def2-TZVP). The plotted surfaces correspond to a surface of 0.005 e·Bohr^{-3}.

Mode	Frequency [cm-1]	Dimensionless Normal Coordinate Displacement
1	348	0.5833
2	625	0.8398
3	736	0.2772
4	943	0.2541
5	1014	0.0582
6	1181	0.4371
7	1332	0.2294
8	1393	0.6602
9	1466	0.2422
10	1518	0.2556
11	1573	0.7738
12	3185	0.0159
13	3202	0.0123

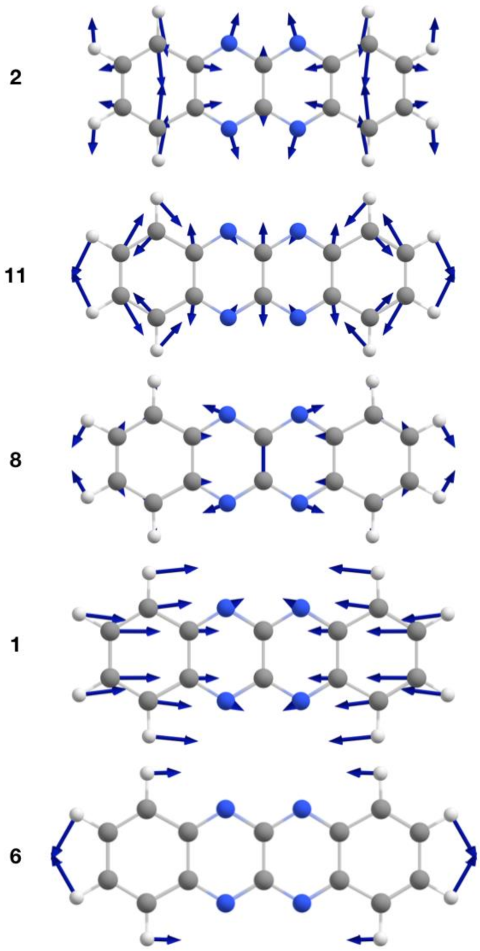

Figure S16: Normal modes and their corresponding displacement vectors with respect to the first excited state above a threshold of 0.0004 to the vibronic spectrum of **3**.

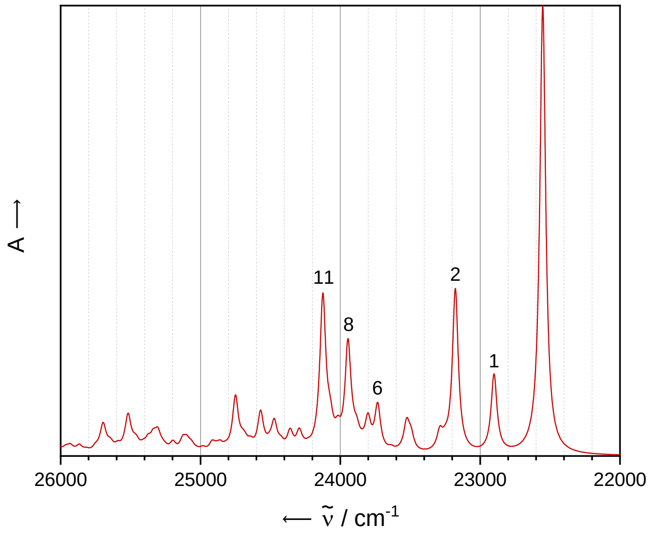

Figure S17: Calculated vibrationally-resolved electronic spectrum of **3** (shifted by 1975 cm^{-1}) with the most essential vibrational modes annotated (B3LYP-D3/def2-TZVP).

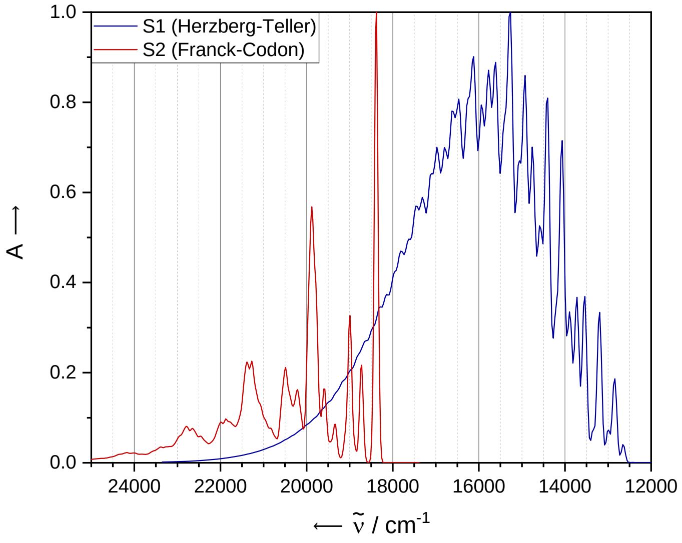

Figure S18: Comparison of the vibrationally-resolved electronic spectra for the S_1 state using the Herzberg-Teller approximation for dipole-forbidden transitions and the S_2 state utilizing the Franck-Condon approximation for dipole-allowed transition of quinoxalino[2,3-*b*]quinoxaline (**3**).

5. High-level calculations of the lowest excited singlet states

5.1. **Tetracene (1)**

Table S19: The three lowest excited singlet states of **1** (ADC(2)/def2-SVPD).

State	Excitation energy / eV	Oscillator strength
B_{2g} (n-π*)	2.53	0
B_{2u} (^1L_b)	3.13	0.06
B_{3u} (^1L_b)	3.53	0.40

5.2. **Benzo[*b*]phenazine (2)**

Table S20: The three lowest excited singlet states of **2** (ADC(2)/def2-SVPD).

State	Excitation energy / eV	Oscillator strength
B_1 (^1L_a)	2.85	0.06
B_2 (n-π*)	3.00	0.00
A_1 (^1L_b)	3.54	0.14

5.3. **Quinoxalino[2,3-*b*]quinoxaline (3)**

Table S21: The three lowest excited singlet states of **3** (ADC(2)/def2-SVPD).

State	Excitation energy / eV	Oscillator strength
B_{2u} (^1L_a)	2.95	0.086
B_{3u} (^1L_b)	3.56	0.00
B_{1g}	4.33	0.00
